# Effectiveness of cone-beam computed tomography-generated cephalograms using artificial intelligence cephalometric analysis

**DOI:** 10.1038/s41598-022-25215-0

**Published:** 2022-11-29

**Authors:** Eun-Ji Chung, Byoung-Eun Yang, In-Young Park, Sangmin Yi, Sung-Woon On, Young-Hee Kim, Sam-Hee Kang, Soo-Hwan Byun

**Affiliations:** 1 Department of Conservative Dentistry, Hallym University Sacred Heart Hospital, Anyang, 14068 Korea; 2 Department of Oral and Maxillofacial Surgery, Hallym University Sacred Heart Hospital, Anyang, 14068 Korea; 3 Department of Orthodontics, Hallym University Sacred Heart Hospital, Anyang, 14068 Korea; 4 Department of Oral and Maxillofacial Radiology, Hallym University Sacred Heart Hospital, Anyang, 14068 Korea; 5 Graduate School of Clinical Dentistry, Hallym University, Chuncheon, 24252 Republic of Korea; 6 Institute of Clinical Dentistry, Hallym University, Chuncheon, 24252 Republic of Korea; 7 Dental Implant Robotic Center, Hallym University Sacred Heart Hospital, Anyang, 14068 Korea; 8 Department of Oral and Maxillofacial Surgery, Hallym University Dongtan Sacred Heart Hospital, Hwaseong, 18450 Korea

**Keywords:** Medical research, Outcomes research

## Abstract

Lateral cephalograms and related analysis constitute representative methods for orthodontic treatment. However, since conventional cephalometric radiographs display a three-dimensional structure on a two-dimensional plane, inaccuracies may be produced when quantitative evaluation is required. Cone-beam computed tomography (CBCT) has minimal image distortion, and important parts can be observed without overlapping. It provides a high-resolution three-dimensional image at a relatively low dose and cost, but still shows a higher dose than a lateral cephalogram. It is especially true for children who are more susceptible to radiation doses and often have difficult diagnoses. A conventional lateral cephalometric radiograph can be obtained by reconstructing the Digital Imaging and Communications in Medicine data obtained from CBCT. This study evaluated the applicability and consistency of lateral cephalograms generated by CBCT using an artificial intelligence analysis program. Group I comprised conventional lateral cephalometric radiographs, group II comprised lateral cephalometric radiographs generated from CBCT using OnDemand 3D, and group III comprised lateral cephalometric radiographs generated from CBCT using Invivo5. All measurements in the three groups showed non-significant results. Therefore, a CBCT scan and artificial intelligence programs are efficient means when performing orthodontic analysis on pediatric or orthodontic patients for orthodontic diagnosis and planning.

## Introduction

Since the introduction of the “new X-ray technique” for cephalometric analysis by Broadbent in 1931, cephalograms have been widely used for measuring the size and shape of craniomaxillofacial structures and evaluating their growth and development^1^. Lateral cephalograms are the representative tool in the evaluation of craniofacial growth, orthodontic diagnosis, treatment planning, assessment of treatment results, and craniofacial growth prediction^2,3^. However, since the conventional cephalometric radiograph displays a three-dimensional (3D) structure on a two-dimensional (2D) plane, it may produce inaccurate results when quantitative evaluation is required. For example, when structures on both sides overlap and have distinct magnifications, it is difficult to distinguish between the left and right sides. This may result in inter-examiner discrepancy and differences according to time between the same inspectors. In addition, depending on the transmission of radiation, the structures in the midsagittal region may have an ambiguous shape, thereby lowering the measurement accuracy in the overlapping structures.

Recently, owing to the innovative development of 3D radiographic techniques, such as cone-beam computed tomography (CBCT), 3D images have been used for orthodontic diagnosis. The indications of CBCT, with some evidence on its clinical efficacy, include impacted teeth, severe craniofacial anomalies, planning and evaluation of combined orthodontic-surgical treatments and bone irregularities, and temporomandibular joint malformation with accompanying signs and symptoms. CBCT has minimal image distortion because there is no difference in magnification according to the region, and important parts can be observed in detail without overlapping images. Furthermore, compared to conventional radiographs, CBCT has a higher resolution and can distinguish between tissues when there is only a difference of 10% in tissue density. In addition, images can be refined using multiplanar reformatting, surface rendering, and volume rendering through computer reconstruction, and evaluation in various directions is possible through image rotation. Clearly, CBCT has advantages over plain lateral cephalometric radiographs, but conventional cephalograms are easier to access than CBCT in many ways. CBCT has been able to address some of the limitations of conventional CT and provides high-resolution images at low radiation dosage and cost, but still exposes patients to greater doses of radiation than conventional lateral cephalometric radiographs^4^. With the rapid improvement in CBCT technology, the gap between accessible scientific data and the lawful use of CBCT is narrowing. This holds especially true for children who are more sensitive to radiation and frequently present with difficult diagnoses^5,6^. The two basic principles of radiological protection of patients should always be followed when considering radiation exposure for diagnostic purposes: justification and optimization^5,7^.

A 2D image, such as the conventional lateral cephalometric radiograph, can be obtained by reconstructing the Digital Imaging and Communications in Medicine (DICOM) data obtained from CBCT. The advantage of this technique is that there is no additional need to record a lateral cephalometric radiograph, skull anteroposterior radiograph, or submentovertex radiograph. Furthermore, when recording a conventional radiograph, the position of the radiographic film is fixed, but in CBCT, the position of the image can be modified using software. In addition, it is possible to reduce the error caused by the magnification of the left and right sides of a conventional 2D image. A CBCT-generated 2D image can be obtained with minimal magnification by using a converting program in the CBCT system.

Lateral cephalogram measurements can be performed manually or with a computer. Manual measurement methods are time-consuming, have a large measurement error, and are greatly affected by the expertise of the operator. In addition, although cephalometric analysis is typically performed by orthodontists trained in clinical practice, there have been many reports of significant intra- and inter-observer variability^8,9^. In computer-assisted cephalometric analysis, computerized cephalometric tracing programs, such as V Ceph (CyberMed, Inc., Seoul, Korea), Rainbow Ceph (Dentium Co, Gyeonggi-do, Korea), and Dolphin Imaging Version 8.0 (Dolphin Imaging, Chatsworth, CA), automatically evaluate the selected landmarks and calculate the distance and angles, thereby reducing inaccuracies that can arise with manual measurement^10^. However, an error might still occur in identifying the landmarks according to the skill level of the examiner in using such software^11^.

Therefore, the need for a fully automated tracing software program to improve the accuracy and reliability of cephalometric measurements is continuously increasing. Artificial intelligence (AI) is widely used in everyday applications. AI-based algorithms are found in almost every technology and used in spam filtering or online voice assistants, internet search engines, and image recognition on social media platforms. Several AI-based programs for automatically identifying anatomical measurement points are being studied currently. These include AI-based orthodontic and orthognathic online platforms, such as WebCeph (Assemble Circle, Gyeonggi-do, Korea), WeDoCeph (Audax, Ljubljana, Slovenia), and Ceph X (ORCA Dental AI, Las Vegas, NV). These are gaining popularity because of their ability to plan orthodontic treatment and obtain patient information quickly. WebCeph includes automated cephalometric tracing, cephalometric analysis, automatic superimposition, visual treatment simulation, photo gallery, and image archiving. Additionally, it enables manual landmark modification and automatic measurement computation.

In this study, conventional lateral cephalograms and lateral cephalograms generated from CBCT data were analyzed using the AI-based landmark measurement program WebCeph. The purpose of this study was to evaluate the applicability of lateral cephalograms generated from CBCT images using an AI-based cephalometric analysis program.

## Results

**Figure 1:**
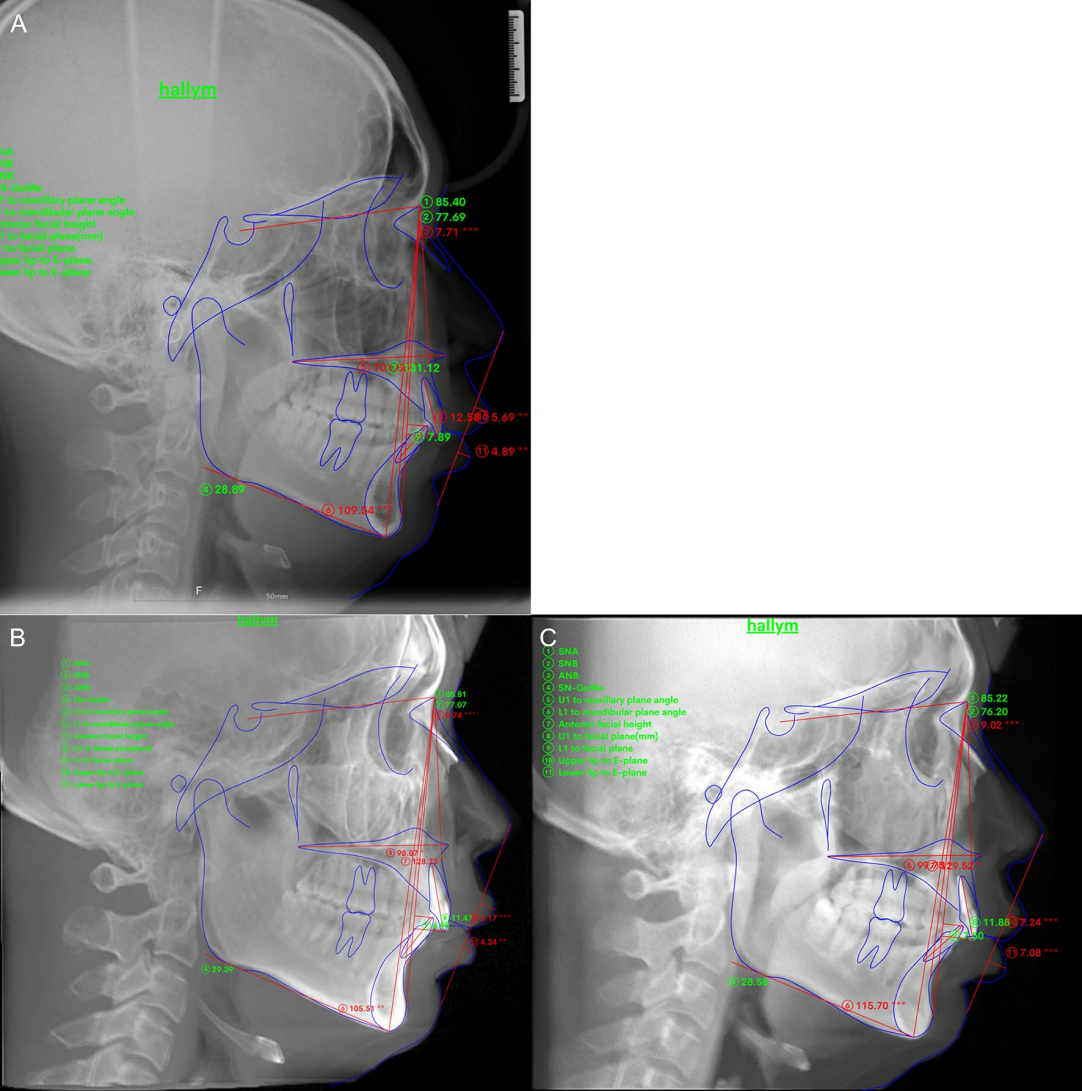
Measurements used in this study. (**A**) Conventional lateral cephalogram, (**B**) lateral cephalogram generated from OnDemand 3D, and (**C**) lateral cephalogram generated from Invivo5.

Cephalometric radiographs of 30 participants (15 male and 15 female) were evaluated. The distribution of skeletal malocclusion was as follows: 13 cases of class I, 14 of class II, and 3 of class III. Figure 1 shows the measurements used in this study. The results of the one-way analysis of variance (ANOVA) are shown in Table 1. In Tukey’s post-hoc test, all measurements were distributed within a 95% confidence interval.

**Table 1:** Assessment of systematic bias between groups I, II, and III using one-way ANOVA.

	Average	Standard error	Standard deviation	Significance
SNA (°)	82.27	3.33	3.33	0.886
SNB (°)	77.67	3.37	3.86	0.657
ANB (°)	4.61	2.03	2.03	0.774
SN-MP (°)	34.09	5.37	5.67	0.754
U1-MaxP (°)	112.33	8.72	8.72	0.910
L1-MP (°)	94.32	5.87	5.87	0.651
N-Me (°)	121.68	9.30	9.30	0.819
U1-NPog (mm)	10.73	4.72	4.72	0.888
L1-NPog (mm)	6.39	3.83	3.83	0.982
Upper lip—E line (mm)	0.443	2.50	2.50	0.461
Lower lip—E line (mm)	2.04	3.11	3.11	0.661

Table 2 shows comparisons of landmark detection between the three groups. When comparing the measured values in 2D cephalograms and regenerated 2D cephalograms from CBCT images, none of the measurements were statistically significant. The maximum differences for the angular measurements were in the L1-MP angle, whereas for the linear measurements, the maximum difference was in the Upper Lip–E line. Between groups I and II, the greatest difference was in the Upper Lip–E line, and the least difference was in the U1-NPog and L1-NPog lines. Between groups I and III, the greatest difference was in the Upper Lip–E line, and the least was in the SN-MP angle. Likewise, between groups II and III, the greatest difference was in the SNB angle, and the least was in L1-NPog.

**Table 2:** Comparisons of landmark detection among the three groups.

	*p* value		
	I-II	I-III	II-III
SNA (°)	0.952	0.981	0.878
SNB (°)	0.804	0.961	0.646
ANB (°)	0.849	0.991	0.779
SN-MP (°)	0.785	1.000	0.797
U1-MaxP (°)	0.984	0.903	0.964
L1-MP (°)	0.872	0.624	0.901
N-Me (°)	0.990	0.820	0.885
U1-NPog (mm)	1.000	0.912	0.901
L1-NPog (mm)	1.000	0.983	0.986
Upper lip—E line (mm)	0.467	0.611	0.969
Lower lip—E line (mm)	0.779	0.661	0.979

*p* value from One-way ANOVA, Tukey’s Post Hoc test.

## Discussion

Conventional CBCT should not be used in general orthodontic practice because of the higher radiation dose. Lateral cephalograms, anteroposterior cephalograms, panoramic radiographs, and temporomandibular joint radiographs, which are usually required to formulate the orthodontic treatment plan, can be generated by our system from a single CBCT session. Although the radiation dose associated with a single CBCT session is higher than that of all the four radiographs combined, as CBCT needs to be performed only once, it can decrease patient discomfort and provide accurate 3D images^12^. Before the widespread use of CBCT, clinicians often missed critical factors such as temporomandibular joint problems, severely impacted tooth, and airway problems^13,14^. As dental clinics now widely use CBCT, many orthodontists and maxillofacial surgeons order CBCT to assess the temporomandibular joint area, position of the third molar, and maxillary sinus for accurate diagnosis. Particularly, if the patient requires orthognathic surgery, CBCT is essential for identifying the position of the anatomic structure. In addition, this study included patients who required both a cephalometric radiograph and CBCT. Therefore, an additional cephalometric radiograph was required when orthodontic analysis was performed in patients who underwent CBCT. If adequate data are obtained with CBCT for orthodontic analysis, additional cephalometric radiographs would not be necessary.

Several studies have been conducted on CBCT use in cephalometry, but most of these studies were on the effectiveness of 3D cephalometric analysis. Three-dimensional cephalometric analysis can be performed using various 3D cephalometric analysis programs. However, most clinicians are familiar with 2D cephalometric analysis, which are mainly based on 2D data. In addition, 3D cephalometric analysis is more complex than 2D cephalometric analysis. Therefore, most clinicians prefer to use 2D cephalometric analysis.

A significant novelty of our study is that it focused on the effectiveness of a CBCT-generated 2D cephalogram, for which there are only a limited number of studies.

The null hypothesis was not rejected based on the statistical analysis results. The lack of significant differences among all evaluated measurements indicates that lateral cephalograms generated from CBCTs are similar to conventional lateral cephalograms. These findings add to the argument that CBCT alone can be used for diagnosis in orthodontics. If there were statistically significant differences, one conclusion would be that lateral cephalograms generated from CBCTs would still be insufficient for clinical use because in this study, the CBCT-generated cephalograms were evaluated on the premise that conventional cephalograms analyzed with AI were correct. For this premise, entire reference points were not compared, and only reliable points were selected and used.

Lateral cephalograms are indispensable for the examination of the relationship between soft tissues, dental tissues, and skeletal structures as well as the diagnosis of anteroposterior and vertical variation in these structures^15^. Therefore, the procedure for cephalometric analysis must be precise, safe, and repeatable. We considered manual cephalometry performed by a single examiner. However, as the 2D cephalogram and CBCT-generated cephalogram images were markedly different, it was impossible to perform a blind test. Thus, AI-based cephalometric analysis may be more objective than manual cephalometry.

Since the development of the first automatic measurement point identification method by Cohen and Linney et al.^16^ in 1984, various studies to improve the automatic measurement point recognition accuracy have been reported, and most of the measurement points showed a high correlation with the measurement results of the examiner. The development of AI has significantly influenced image analysis, particularly medical image analysis^17^. Several algorithms have been developed to automatically recognize these anatomical indicators using various AI models, and dentistry is no exception. These algorithms enable inexperienced clinicians to consistently detect landmark points and analyze them. The AI of WebCeph uses a deep learning algorithm. The deep learning algorithm uses a convolutional filter and pooling layer to extract features from an image and analyze the patterns. Filter sizes, regions, categorization, combinations, and so on have been used to enhance and develop many deep learning models. Since they leverage spatially on local correlation by enforcing local connection patterns, convolution neural networks are particularly well-suited for image processing and recognition applications. Consequently, it is expected that when the diagnostic image data are evaluated using deep learning, the empirical knowledge gained from examining the image data would be better reflected. Clinicians appreciate time-saving and convenience of use as two of the many benefits of digital cephalometry. Measurement reproducibility is required to determine the accuracy of any method of analysis. The use of computers in treatment planning is predicted to eliminate the incidence of errors caused by fatigued operators and offer a uniform, quick, and effective evaluation with a high rate of repeatability^18^. According to recent studies, AI can identify landmarks as accurately as human examiners, and it might be a viable choice for repeated recognition of numerous cephalometric landmarks^19^. The successful detection rates of 19 skeletal landmarks with a 2-mm range^20–22^, which has usually been acknowledged as a clinical error range in AI performance^23^, have traditionally been used to compare the performance of an automated identification system.

In general, differences between the three groups were not statistically significant in any of the assessments. Differences in the linear measurements were larger than changes in the angular measurements, possibly because of image distortion or calibration. This is in line with the findings of a previous study^15^. According to the study by Chen et al.^18^, the menton, gonion, lower incisor apex, orbitale, and porion are the most questionable and unpredictable points irrespective of the method utilized for locating the landmarks. In addition, Lagravere et al.^24^ reported that the menton, nasion, and posterior nasal spine also result in errors. Hwang et al.^25^ described that the orbitale and PNS displayed higher standard deviation values when detected by AI because such landmarks are difficult to recognize owing to overlapping cranial base structures.

The accuracy depends on the size of the training dataset, which increases as the number of landmarks to be detected increases^26^. Most studies evaluating these software programs used lateral cephalometric radiographs obtained from a small number of cephalography equipment^27^. In the present study, WebCeph was used to evaluate 2D conventional lateral cephalograms obtained from specific devices, not lateral cephalograms generated from CBCT. WebCeph could not identify the landmark around the mandibular symphysis area, which could have affected the results of this study. This may be because the anterior region of the mandible of each patient was stabilized by a chin holder when CBCT was acquired. Apart from the inability to locate a specific point, landmarks may not be identified in more cases using WebCeph depending on what kind of radiographic device was used. In some cases, WebCeph deviates completely from a specific location, especially in the mandibular symphysis area. The widest possible failure category in training the software is the mandibular symphysis.

In summary, most inconsistencies were observed in linear measurements. The findings of the present study showed that tracing with the automatic WebCeph resulted in obvious inaccuracies, such as in landmark detection, where points were frequently identified outside the bone or at the wrong location; in soft tissue outline tracing, where the tracing line was clearly drawn away from the soft tissue outline; and in the detection of the average of bilateral points. These are all significant concerns that can have a direct influence on the analysis outcome and were identified in all the radiographs examined in this study.

Another factor that influenced the results of this study was the use of 2D radiographs generated from CBCT for analysis. Compared with conventional cephalograms, during image acquisition, the errors due to faulty positioning of the patients could be modified in CBCT datasets by repeated correction and reassessment. The inherent 3D properties of the CBCT dataset enable the generation of an endless number of reformatted images and orthogonal cephalograms^28,29^. Moreover, it is possible to represent both sides of the skull, preventing the superimposition of the left and right structures. However, the development of 2D skull landmarks and virtual 3D CBCT models remains an obstacle^30^. Owing to the characteristics of the 3D anatomical structures, landmarks are often missing in 2D. In 3D images, the acute edges observed in conventional lateral cephalograms are replaced by surfaces and curves. For example, the ear rods used in conventional cephalograms point to the location of the external auditory meatus; however, the anatomic porion differs from the external opening. According to van Vlijmen et al.^31^, the sella, upper incisor apex, incision inferius, and lower incisal apex are difficult to recognize using the 3D model. Since all these points are contained within the 3D model, CBCT slices should be selected to be able to designate their position^32^. However, several in-vitro and in-vivo investigations found no difference^29–31^.

Since the landmark measurements obtained from the CBCT-generated images are comparable with the virtual distances and angles between skull locations and the measurements made on conventional lateral cephalograms, the need for further conventional lateral cephalograms can be reduced, thereby avoiding additional radiation exposure to the patient. In addition, AI in CBCT analysis will be a beneficial addition and should be studied further in future research^33^. We are in the process of developing low-dose CBCT using various algorithms to reduce exposure rate. We plan to evaluate the significance of AI-based low-dose CBCT acquisition in orthodontic diagnosis. The present study could act as a starting point for a CBCT-based orthodontic diagnosis system.

Cephalograms generated from CBCT should be used by recognizing their limitations and considering the advantages in terms of radiation dose, convenience, and cost. In addition, through the development of AI and integration of CBCT, it can be expected that orthodontic diagnosis and treatment planning will be easier in the future.

## Methods

### Study participants

CBCT and lateral cephalograms were recorded for patients who visited the Department of Dentistry, Hallym University Sacred Heart Hospital. The study participants comprised 15 male and 15 female patients, with a mean age of 16.57 years and an age range of 7–41 years. Informed consent was obtained from all subjects involved in the study. Specific consent was obtained to publish the images of participants in an online open-access publication. Written informed consent has been obtained from a patient and/or legal guardian for minors to publish this paper. The inclusion criteria were as follows:

- A.Patients with systemic diseases that were medically well-controlled
- B.Patients who required cephalometric radiograph and CBCT
- C.Patients without any maxillofacial deformity
- D.Patients with erupted incisors and first molars

Patients for whom radiographs could not be recorded were excluded from the study. The radiographs were categorized into three groups. Group I included conventional lateral cephalograms, group II included cephalometric radiographs generated from CBCTs using OnDemand 3D (Cybermed Co., Seoul, Korea), and group III included cephalometric radiographs generated from CBCTs using Invivo5 (KaVo Co., Biberach, Germany). The study was conducted according to the guidelines of the Declaration of Helsinki and approved by the Institutional Review Board of Hallym University Sacred Heart Hospital (IRB Approval No. 2021-07-016-005). In the entire research, the personal information of the patients was not disclosed.

## Materials

### CBCT Protocol

CBCT scans were recorded using Alphard Vega (Asahi Roentgen Inc., Kyoto, Japan), with a slice thickness of 0.39 mm, 3 voxel size level, 20.0 × 17.9 cm exposure area, 4 mA, 80 kV, and 17 s exposure time. The collected data were imported into OnDemand 3D and Invivo5 as DICOM files.

### Lateral cephalometric radiograph protocol

Lateral cephalograms were obtained using Rayscan Alpha (Ray Co., Gyeonggi-do, Korea). During imaging, both ear rods of the head restraint were inserted into the participant’s ear hole, and the head was fixed. During imaging, the tube current was 4–17 mA, tube voltage was 60–90 kV, and exposure time was 3.8–9.9 s (group I). The radiographs were saved as JPEG files for easy comparison with groups II and III.

### Generation of lateral cephalometric radiographs from CBCT data

After recording the CBCT, the stored DICOM file was reconstructed into 2D lateral cephalograms using the X-ray generation module in the OnDemand 3D and Invivo5 programs. The midsagittal plane of the patients was aligned vertically using the axial view, the transporionic line was positioned horizontally using the coronal view, and the Frankfort plane was oriented horizontally using the sagittal perspective. The reconstructed images were saved as JPEG files**.**

### Landmark identification

**Figure 2:**
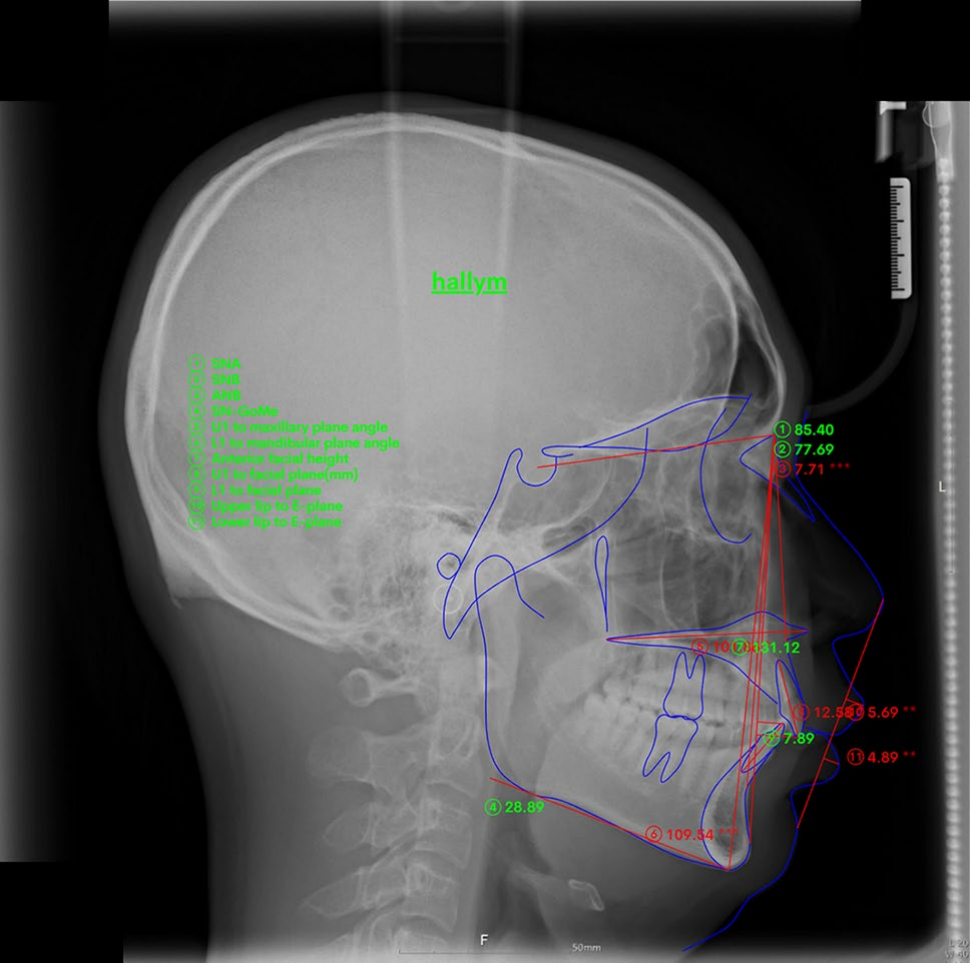
Major landmarks used in this study with AI-based tracing program.

Conventional lateral cephalometric radiographs and lateral cephalometric radiographs generated from the CBCT data were automatically measured using Webceph. Figure 2 shows the automatic tracing of the measurement points in WebCeph using AI. Each analyzed image was saved individually. On each lateral cephalometric radiograph, 17 measurement points were indicated, and 11 measurements representing the skeletal, dental, and soft-tissue characteristics were evaluated, including six angular and five linear measurements. The bilateral structures were averaged to create a single measurement point. The measurements used in the study were as follows: SNA (°), SNB (°), ANB (°), SN-MP (°), U1-MaxP (°), L1-MP (°), N-Me (mm), U1-NPog (mm), L1-NPog (mm), upper lip-E line (mm), and lower lip-E line (mm).

### Statistical analysis

Statistical analysis was performed using Statistical Package for Social Sciences for Windows (version 25.0; SPSS Inc., Chicago, Illinois, USA). The data are presented as the mean, standard error, standard deviation, and significance values. To compare the differences in measured values between the three groups, a one-way ANOVA and Tukey’s post hoc test were used. The significance level was set at *p* < 0.05, and the results of the study group were estimated with a 95% confidence interval.

## Data Availability

The datasets used and/or analyzed during the current study available from the corresponding author on reasonable request.
